# 

**Published:** 1891-06-06

**Authors:** 


					Iflli
HOMAS'S UoWpt
IRYf/CH HOSPl TAL
.ASOO W WES TERN INFIRMARY
WITH EXTRA NURSING SUPPJ EMENT.
OVRIL
J'mM H ?? ? ? in the residue thev a? XM V the Extract of Meat, the Albuminous prmoiples remain
? ? 11 wm "BOVBII." contains rir^ B' l JkNJ liBt & nntrition, and this is certainly a great disadvantage.-
requirements of the human system and the * \1\ JIT!! /iSl\ most P6^Possible form, and to
Identical with what the body requires for recuperation, )1L t*?S> M) JMCy . \ Llf i i WlU appar.enti^at ^is albumen and
?ny animal aliment at Dresent known. and that as a perfect form of concentrated nourishment it mturt
P SOLD THROUGHOUT THE UNITED KINGDOM.
No. 245. Vol. X."
Saturday,
"June 6th, 1891.
[One Penny.
X -

				

